# Gene Mutations in Lung Cancer: Promising Predictive Factors for the Success of Molecular Therapy

**DOI:** 10.1371/journal.pmed.0020013

**Published:** 2005-01-25

**Authors:** Akira Inoue, Toshihiro Nukiwa

## Abstract

Gefitinib and erlotinib are two new treatments for advanced lung cancer. Gene mutations in the cancer may help predict which patients will respond to these treatments

Lung cancer is the leading cause of death in many countries. To date, cause of cancer death in chemotherapy with cytotoxic agents has been the mainstay of treatment for advanced lung cancer. However, the activity of these agents is quite limited, and they have severe adverse effects. Recent, rapid advances in molecular biology have led to the development of many new agents that inhibit the activities of specific molecules related to tumor growth, invasion, or metastasis [1], and these agents have the potential to improve the outcome of lung cancer treatment. But we have not yet managed to successfully deliver these agents from the bench to the bedside.

## Molecular Therapy for Lung Cancer

Gefitinib is the first “molecular-target agent” for lung cancer that inhibits the tyrosine kinase of the epidermal growth factor receptor (EGFR; also known as ERBB1). EGFR is frequently overexpressed in non-small-cell lung cancer (NSCLC), especially in squamous cell carcinoma, and its expression is related to the cancer's proliferation. Initial clinical trials of gefitinib showed its modest clinical activity in patients who had failed previous standard chemotherapy. But subsequent randomized trials in patients with previously untreated, advanced NSCLC have not shown a clinical advantage of gefitinib combined with standard chemotherapy over chemotherapy alone [2,3,4,5].

Erlotinib, another EGFR tyrosine kinase inhibitor for treating NSCLC, which was approved by the United States Food and Drug Administration in November 2004, has a therapeutic profile similar to that of gefitinib. Erlotinib has shown a survival benefit in a phase III trial for chemo-refractory NSCLC that compared erlotinib to best supportive care [6]. (To date, there have been no trials showing a survival benefit of EGFR inhibitors over standard chemotherapy.) Interestingly, in these trials of EGFR inhibitors, EGFR expression levels in the tumors were not correlated with the response; high response rates were seen in women, patients with adenocarcinoma, nonsmokers, and Japanese patients. Recent studies have now shed light on why certain patients are more likely to respond than others—the key lies in the presence of gene mutations.

## EGFR Gene Mutations in NSCLC

In April and May 2004, two new studies published in *Science* and the *New England Journal of Medicine* helped to explain at the molecular level the low clinical activity of EGFR inhibitors [7,8]. These studies identified somatic mutations in the EGFR gene, especially around the region encoding the ATP-binding pocket of the receptor's tyrosine kinase domain. These mutations increased the sensitivity of tumor cells to gefitinib. A high incidence of mutations was detected in patients with NSCLC that had a durable clinical response to gefitinib, and subsequent studies revealed that these mutations were also related to the response to erlotinib. Moreover, such mutations were more frequently detected in patients with adenocarcinoma, in women, in Japanese patients, and in nonsmokers [9]—results that were compatible with previous clinical data.

These findings should hopefully lead to the identification of subgroups of patients who are likely to benefit substantially from such EGFR inhibitors. But another question was raised by these studies: is it only EGFR gene mutations that determine the response to EGFR inhibitors?

## RAS Gene Mutations in NSCLC

**Figure 1:**
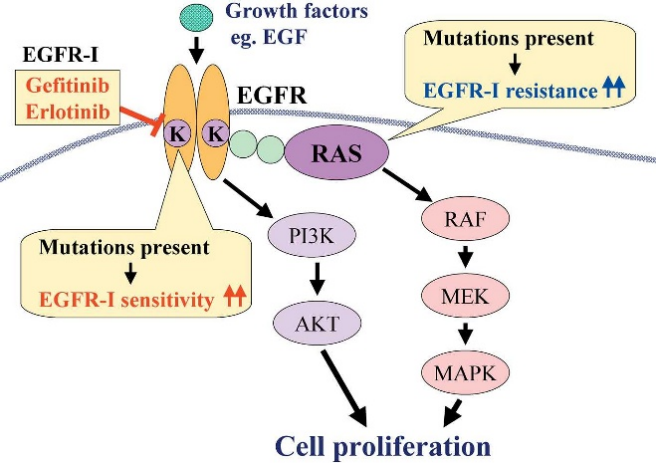
EGFR Signal Transduction in Cancer Cells Arrows indicate stimulation, and T-bars, inhibition. EGFR-I, EGFR inhibitor; MEK, MAPK kinase.

The RAS proteins are low-molecular-weight GTPases that are bound to the inner side of the cell membrane. They are involved in signal transduction pathways: they regulate downstream effector proteins such as Raf/MAP kinase and PI3 kinase, under the influence of various cell surface receptors including EGFR (Figure 1). Mutations of the K-ras gene have been found in up to 30% of lung adenocarcinomas and have been considered a poor prognostic factor [10]. A study by Pao and colleagues published in this issue of *PLoS Medicine* suggests that the K-ras mutation is an important predictive factor in defining which patients will benefit from receiving EGFR inhibitors [11]. In Pao and colleagues' study, the K-ras mutations were completely associated with a lack of response to EGFR inhibitors (0/14 tumors with K-ras mutations were sensitive). The EGFR mutations were significantly related to response (17/17 tumors with EGFR mutations were sensitive), as observed in previous studies.

Although the number of tumors examined in this study may be too small to lead to a definite conclusion—and, furthermore, about half of tumors were retrospectively collected—this study is the first to show that mutations of EGFR and K-ras are not related and that K-ras mutations are associated with a lack of sensitivity to EGFR inhibitors. However, since the sensitivity of the method for finding each mutation influences how often it is detected, standardization of detection methods is important. Hence, the true incidence of K-ras mutations in NSCLC (including non-adenocarcinoma) and their refractoriness to EGFR inhibitors need to be established in further studies. In fact, the reported incidence of EGFR mutations differs between institutions. In addition, although Pao and colleagues' study examined such mutations only by DNA sequence, mRNA or protein expression would show mutation status more accurately.

## Therapeutic Implications

In the clinical setting, prolonged disease stabilization with no measurable reduction in tumor size is seen in about half of patients treated with EGFR inhibitors, and a significant survival benefit in this group was shown in a phase III trial [6]. The mutation status of the EGFR gene and K-ras gene among those stabilized tumors should be evaluated; it may reveal two different groups differentiated by mutation status.

It will also be important to establish whether, in tumors with an EGFR mutation that eventually acquire resistance to EGFR inhibitors, the resistance is associated with a change of EGFR status (mutation or change in expression) or is associated with a change of K-ras status. Such changes can be revealed by re-examination of tumors at the time of relapse. If resistance is K-ras dependent, the new “K-ras inhibitors” (unfortunately not yet available) may be of help for patients who have developed a K-ras mutation, as well as for patients whose tumors harbor K-ras mutations from the beginning.

Although many issues still need to be resolved step by step through prospective trials to show the benefit of this new strategy, these mutations are promising predictive factors for the success of EGFR inhibitors. Even if the population who may benefit from EGFR inhibitors (such as patients who are positive for an EGFR mutation and negative for a K-ras mutation) is very small, the response rate of over 80% is encouraging, and has never before been achieved in advanced NSCLC. Finally, it is important to detect which patients derive no benefit from EGFR inhibitors because severe adverse effects such as acute lung injury can occur in any patient treated with these drugs [12]. By combining all the factors that relate to response or resistance, patients who will benefit from treatment can hopefully be identified. Undoubtedly we have taken a great step forward in molecular therapy for lung cancer treatment.

## Abbreviations

EGFR: epidermal growth factor receptor
NSCLC: non-small-cell lung cancer
